# Food and the Principles of Dietetics

**Published:** 1911-07

**Authors:** 


**Food and the Principles of Dietetics.** By **Robert Hutchinson,** M.D., Edin., F.R.C.P., Physician to the London Hospital; Physician with charge of Out-Patients to the Hospital for Sick Children, Great Ormond Street. Third edition. Octavo, pp. 615. With plates and diagrams. New York: William Wood & Co. 1911. (Cloth, $3.00.)

Hutchinson's work has become quite well known through earlier editions. One of the most interesting personal ideas contained in it, is that of dividing the picture of a cabbage, egg, banana, nut, etc., so as to indicate the percentage composition in proteid, fat, carbohydrate, water, etc. Almost every conceivable item in regard to food stuffs and therapeutics of individual diseases is systematically considered but the comprehensive analytic tables of several well known American dietetics, are lacking so that for reference study, the writer will at times be subject to inconvenience. In a few instances, too, as in the consideration of biscuits and the caloric value per unit of cost, it must not be forgotten that we are dealing with English definitions and standards.

The special feature of Hutchinson's book, in our opinion, is one which it is impossible to illustrate in a brief review. Almost every section contains something unexpected in the way of historic interest, or an allusion to chemistry, physiology, clinical phenomena and the like—something which would not have been missed if it had been omitted and yet which is worth knowing and which sets the reader thinking. Thus, the book is one to read, not to place on the shelf for an occasional reference and it has a literary charm and sustains the interest of the reader very much as does a well written book of travel, yet without descending from the plane of serious, technical study. This feature can be appreciated only by perusing the book itself.

